# Elevated potassium levels suppress T cell activation within tumors

**DOI:** 10.1186/2051-1426-3-S2-P403

**Published:** 2015-11-04

**Authors:** Robert L Eil, Rahul Roychoudhuri, David Clever, Shashank Patel, Madhu Sukumar, Jenny H Pan, Douglas Palmer, Christopher A Klebanoff, Nicholas P Restifo

**Affiliations:** 1 NIH/NCI - Surgery Branch, Bethesda, MD, USA; 2 Center for Cancer Research, NCI/NIH, Bethesda, MD, USA

## 

Tumors progress in immunocompetent hosts despite the ability of the adaptive immune system to recognize cancer cells. Ion gradients regulate T cell function but their role in intratumoral immune responses is unexplored. We found that the concentration of K^+^ was strikingly elevated within tumors while the concentration of the divalent cations Ca^2+^ and Mg^2+^ was similar to serum levels. High K^+^ levels significantly blunted cytokine production and suppression TCR stimulation induced gene transcription in CD8^+^ and CD4^+^ effector T cells. Moreover, polarization of CD8^+^ and CD4^+^ T cells in high K^+^ suppressed effector differentiation and promoted the formation of CD4^+^ Foxp3^+^ T_reg_ cells. Surprisingly, this was not due to an attenuation of TCR induced Ca^2+^ flux, but rather to reduced activation of the serine/threonine Akt-mTOR pathway and could be partially reversed by overexpression of constitutively active Akt1. This coincided with the finding that okadaic acid, an inhibitor of the serine/threonine phosphatase PP2A, rendered effector cells resistant to the inhibitory effects of high K^+^ and restored cytokine function within tumors. Additionally, expression of a peptide inhibitor targeting the PP2A complex provided resistance to the inhibitory effect of elevated K^+^. These findings identify a novel mechanism of ionic regulation of TCR induced signals and immunosuppression within tumors whereby locally high extracellular concentrations of normally intracellular ions suppress immune function to promote tumor growth.

